# Myopericarditis Secondary to Toxoplasma Gondii Infection in an Immunocompetent Young Male—A Case Report

**DOI:** 10.3390/reports9020192

**Published:** 2026-06-20

**Authors:** Niall Leahy, Sandra Quinn, Derek Crinion

**Affiliations:** St. James’s Hospital, D08 NHY1 Dublin, Ireland

**Keywords:** toxoplasma, myopericarditis, immunocompetent, guidelines, case report

## Abstract

**Background and Clinical Significance**: Inflammatory myopericardial syndrome is an umbrella term recently introduced by the European Society of Cardiology, which encapsulates the overlap that exists in clinical practice between myocardial and pericardial disease. It has a heterogeneous aetiology and a broad spectrum of severity in terms of its clinical features. Toxoplasma gondii is a rare but recognised infectious cause of myopericarditis and is typically seen in immunocompromised individuals. **Case Presentation**: We present the case of a young, immunocompetent male, presenting with pleuritic chest pain following a recent flu-like illness. Investigations revealed an acute myocardial injury based on elevated troponin T levels, in the absence of ventricular dysfunction. Toxoplasma immunoserology was consistent with primary toxoplasma infection. The remainder of his viral panel was negative. There was prompt symptom improvement following commencement of treatment with colchicine and a non-steroidal anti-inflammatory agent. Cardiac magnetic resonance imaging post-discharge revealed findings consistent with prior myocarditis. **Conclusions**: This case is an example of the rare occurrence of toxoplasma myopericarditis in an immunocompetent individual. Cardiac MRI is an invaluable imaging modality used to evaluate myocardial function and tissue characteristics in patients presenting with inflammatory myopericardial syndrome.

## 1. Introduction and Clinical Significance

Inflammatory myopericardial syndrome (IMPS) is a term recently introduced in the European Society of Cardiology (ESC) guidelines for the management of myocarditis and pericarditis [1]. This umbrella term succinctly encompasses the overlap that exists between myocarditis and pericarditis in clinical practice.

The aetiology of myocarditis and pericarditis is mostly similar and broadly includes infectious and non-infectious causes [2]. Toxoplasmosis is a communicable disease caused by Toxoplasma gondii, a protozoan parasite, and commonly occurs in tropical and subtropical countries across the world [3]. Infection in immunocompetent individuals typically results in mild and self-resolving symptoms, and many patients are asymptomatic, but in immunocompromised patients, it can result in more severe clinical presentations [3,4]. Toxoplasma gondii infection is an uncommon cause of myopericarditis and typically occurs in immunocompromised individuals. We present a case of it occurring in an immunocompetent male and discuss some of the considerations involved in the work-up and management of this condition.

## 2. Case Presentation

A twenty-eight-year-old male presented to the Emergency Department with a three-day history of chest pain radiating to his left arm. The pain was sharp in character and exacerbated with lying flat, and relieved with sitting forward. Prior to the onset of chest pain, he reported a seven-day history of a flu-like illness, consisting of symptoms including fever, cough, malaise, and myalgia. The patient had no other past medical history of note and was not on regular medications. He was a non-smoker and seldom consumed alcohol. He denied any recent foreign travel or recreational drug use. He did not keep any pets, although he reported passive exposure to a cat living in the same residential facility as him. There was no significant family history.

On presentation, he was haemodynamically stable, with a non-invasive blood pressure of 111/74 mmHg and a heart rate of 77 beats per minute. He was apyrexic and oxygen saturation was normal. On clinical examination, dual heart sounds were audible with no murmurs or pericardial rub appreciated, and there were no clinical features of decompensated heart failure. The remainder of his physical examination was unremarkable.

Baseline laboratory investigations with abnormal results were as follows: high sensitivity cardiac troponin T (hs-cTnT) 525 ng/L (reference range < 14 ng/L), high sensitivity C-reactive protein (CRP) 56 mg/L (reference range 0–5 mg/L), N-terminal pro-brain natriuretic peptide (NT-pro BNP) 250 pg/mL (reference range 125 pg/mL). Additional laboratory investigations, including full blood count, renal function, liver function, and serum electrolytes, were all within normal range. Chest x-ray showed no acute pathology.

12-lead electrocardiogram (ECG) showed normal sinus rhythm with a narrow QRS complex and normal PR and QT intervals. There was less than 0.5 mm ST elevation in V5, V6, lead I, lead II, and lead aVL, and subtle PR depression was present (Figure 1).

The initial working diagnosis was myopericarditis. He was admitted for further investigations and management, and was commenced on continuous cardiac monitoring. Evaluation for SARS-CoV-2 virus, influenza, and respiratory syncytial virus (RSV) was negative. Following consultation with Microbiology, an extended viral panel including hepatitis A, B, and C, herpes simplex virus 1 and 2, human immunodeficiency virus (HIV), parvovirus B19, cytomegalovirus, and Epstein–Barr virus, was negative. In addition, Toxoplasma gondii serology was requested due to the patient having a recent exposure to cats. The patient was commenced on a non-steroidal anti-inflammatory agent, as well as colchicine 0.5 mg twice daily.

Transthoracic echocardiogram performed on day 1 of admission revealed normal biventricular size and function, with no evidence of regional wall motion abnormalities. There were no valvular abnormalities or significant pericardial effusion (Figure 2).

Continuous cardiac monitoring did not demonstrate any transient or sustained arrhythmias. Peak troponin level rose to 647 ng/L, with subsequent down-trending observed on serial assessment. The patient had a prompt and significant symptom improvement following commencement of treatment and was discharged 5 days post-admission.

Results of the Toxoplasma immunoserology testing returned one week post-admission. This showed positive Toxoplasma IgM (125 IU/mL, rising to 500 IU/mL on repeat testing) and negative IgG. Repeat serological testing performed 9 days later showed positive Toxoplasma IgG, confirming seroconversion, thus consistent with primary Toxoplasma gondii infection. Confirmatory serological testing was performed in the Toxoplasmosis reference laboratory in the UK, following this, which confirmed the initial findings.

Cardiac magnetic resonance imaging (MRI) was performed 12 weeks post-discharge. This demonstrated preserved biventricular systolic function. T2 mapping did not demonstrate changes reflective of interstitial oedema. Late gadolinium enhancement sequences demonstrated subepicardial fibrosis in the basal to mid anterolateral and inferolateral segments of the left ventricle, likely reflective of previous myocarditis. The pericardium was normal without evidence of pericardial thickening or enhancement, with a trace of pericardial fluid adjacent to the left ventricular lateral wall (Figure 3).

The overall unifying diagnosis for the case was primary toxoplasma myopericarditis. On his most recent review in the Cardiology outpatient clinic, the patient reported being asymptomatic and is back engaging in physical activity. Following discussion with the local Infectious Diseases team and the Toxoplasmosis Reference Laboratory in the UK, specific treatment for the infection was not commenced, owing to his clinical recovery and symptom resolution. He is planned for ongoing follow-up in the Cardiology clinic.

## 3. Discussion

Amongst the spectrum of inflammatory diseases included under the term IMPS, including isolated myocarditis and isolated pericarditis, myopericarditis represents the most precise diagnosis in this case, as the patient had definite criteria for pericarditis (typical symptoms in addition to PR depression on ECG and elevated CRP) and elevated biomarkers of myocardial injury, in the absence of regional or global impairment of left ventricular function on echo or cardiac MRI [1].

Regarding the clinical presentation of acute myocarditis specifically, it encompasses significant variation and can range from mild symptoms with minimal ventricular dysfunction to fulminant heart failure and cardiogenic shock [5]. Its true incidence has likely been underestimated due to the absence of widespread cardiac MRI use [6]. Historically, endomyocardial biopsy (EMB) has been the gold standard for the identification of myocarditis, especially in the context of new-onset unexplained heart failure, albeit not without procedure-related risks which can range from 0.6% to 5% depending on the setting and centre experience [7,8]. The new guidelines place a stronger emphasis on multimodality imaging, namely cardiac MRI, in the diagnostic process. Importantly, they outline that a definite diagnosis of myocarditis can be made with an appropriate clinical presentation, and either a cardiac MRI-proven or EMB-proven result (1). In cases of myocarditis with ‘high-risk clinical features’, such as cardiogenic shock, and in cases when knowledge of the histological subtype is important for targeted therapies, EMB is recommended [1].

Cardiovascular involvement in toxoplasmosis is rare and often asymptomatic. The extent of the literature discussing cardiac toxoplasmosis is small and mainly comprises cases involving immunocompromised individuals [3]. When cardiac involvement is seen, it typically manifests as myocarditis, which is characterised by inflammatory cell infiltrate, with or without myocyte necrosis [9]. Pericardial involvement can also occur [10]. Similar to myocarditis secondary to other causes, the clinical course can range from a mild and self-limiting illness to fulminant disease resulting in severe left ventricular impairment necessitating haemodynamic support [11].

Treatment of toxoplasma myocarditis in immunocompetent patients with mild symptoms, as seen in this case, typically does not require specific treatment. However, in more severe cases resulting in organ dysfunction, as may be seen in immunosuppressed patients, treatment with pyrimethamine plus sulfadiazine and folinic acid is the standard regimen used [12].

Prognosis varies based on the severity of the clinical presentation. Low-risk myocarditis, which accounts for the majority of presentations (including this case) and is typically characterised by a chest pain presentation in the absence of significant ventricular dysfunction and/or arrhythmias, carries a good short- and long-term prognosis. The finding of late gadolinium enhancement on cardiac MRI in this case is an important prognostic factor, albeit the overall burden in this patient was low. Ongoing outpatient surveillance with cardiac MRI, ECG, and Holter monitor is planned, in accordance with current European guidelines [1].

An important limitation in this case was the absence of a cardiac MRI performed during the patient’s inpatient course. This would have enabled demonstration of active myocardial inflammation in the acute phase of the disease. However, due to difficulty with timely access to inpatient cardiac MRI in our institution, in addition to the patient’s prompt symptom improvement, it was performed on an outpatient basis instead. This subsequently demonstrated a pattern of myocardial fibrosis consistent with prior myocarditis. Another limitation of this case is that polymerase chain reaction (PCR) testing of cardiac tissue or an EMB was not performed; thus, the causal link between Toxoplasma gondii infection and the observed myocardial injury remains speculative. However, as noted above, performing cardiac biopsy should be reserved for ‘high-risk’ cases owing to the invasive nature of the procedure and associated risks. Our patient did not have ‘high-risk’ features, and therefore, there was no strong clinical justification to perform a biopsy. The serological investigations clearly demonstrated active Toxoplasma gondii infection at the time of the patient’s clinical presentation, which was consistent with myopericarditis. Subsequent cardiac MRI demonstrated a prior myocardial injury pattern consistent with myocarditis, and therefore, we feel that based on the totality of this evidence, the diagnosis in this case is justified.

## 4. Conclusions

Cardiac involvement in acute toxoplasmosis is rare but well-documented and typically occurs in immunocompromised individuals. This case adds to the literature regarding occurrence in an immunocompetent host, which to date, has only been reported in a few cases in the last few decades [4,13,14,15]. Cardiac MRI is an invaluable imaging modality used to evaluate myocardial function and tissue characteristics and has a class 1 recommendation in the ESC guidelines for suspected myocarditis [1]. The clinical course of myopericarditis secondary to toxoplasma is typically mild, but clinicians ought to be aware of the spectrum of cardiac manifestations and to instigate appropriate treatment when necessary.

## Figures and Tables

**Figure 1:**
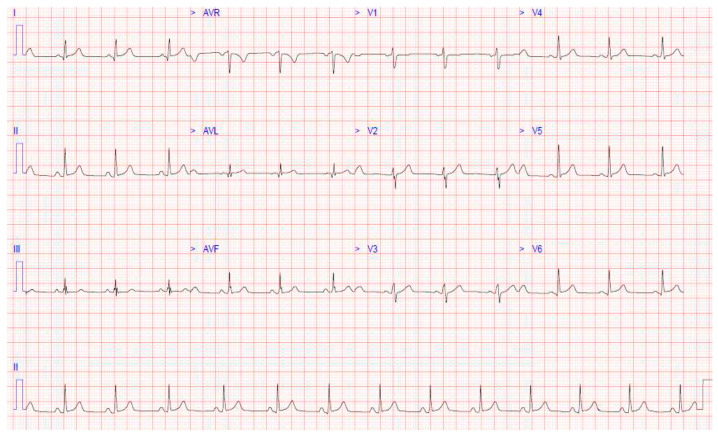
12-lead ECG at the time of admission showing widespread ST elevation and subtle PR depression.

**Figure 2:**
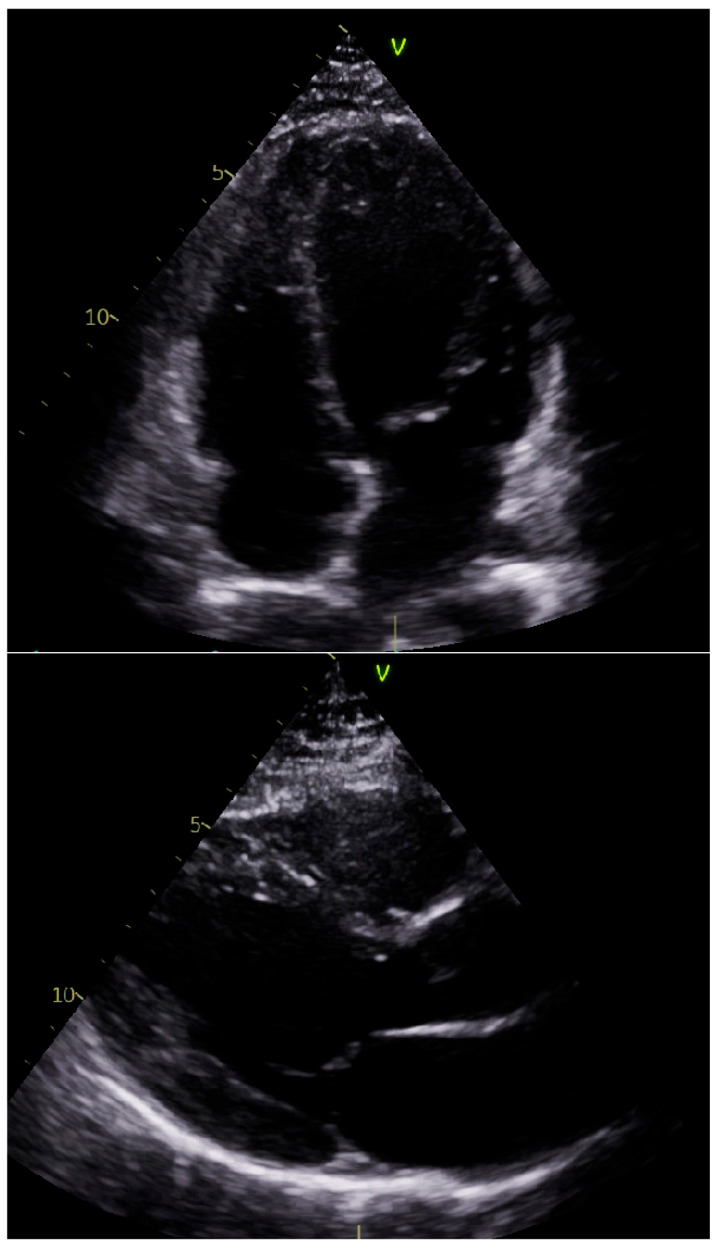
Still images from the patient’s transthoracic echocardiogram. The apical four-chamber and parasternal long axis views in the top and bottom panels, respectively, demonstrate normal biventricular size and no evidence of significant pericardial disease.

**Figure 3:**
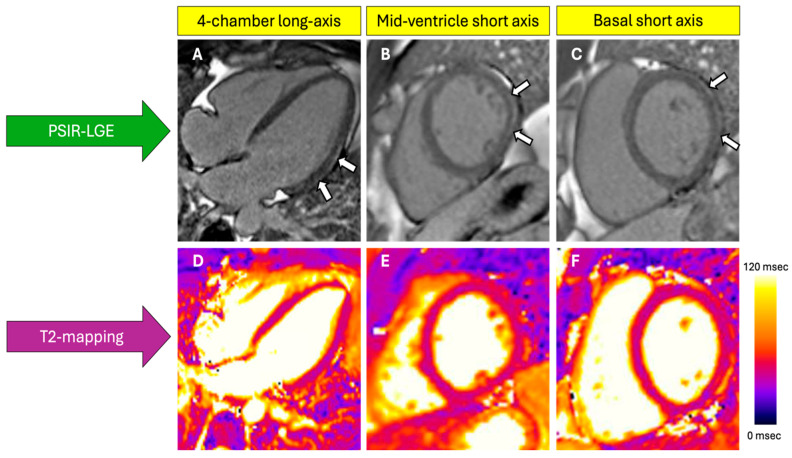
Cardiac MRI performed 98 days after clinical diagnosis of myopericarditis in a 28-year-old immunocompetent male. Phase-sensitive inversion recovery (PSIR) late gadolinium enhancement (LGE) sequences (**A**–**C**) demonstrate subepicardial enhancement (white arrows) in the basal-to-mid anterolateral and basal-to-mid inferolateral segments consistent with non-vascular myocardial injury. Balanced steady-state free precession (bSSFP) T2 mapping (**D**–**F**) values were normal at the time of imaging (mean 49 msec), suggesting no active oedema or inflammation at the time of the cardiac MRI.

## Data Availability

The original data presented in the study are included in the article; further inquiries can be directed to the corresponding author.

## Abbreviations

The following abbreviations are used in this manuscript:

IMPS	Inflammatory myopericardial syndrome
ESC	European Society of Cardiology
hs-cTnT	high sensitivity cardiac troponin T
CRP	C-reactive protein
NT-pro BNP	N-terminal pro-brain natriuretic peptide
ECG	Electrocardiogram
RSV	Respiratory syncytial virus
HIV	Human immunodeficiency virus
MRI	Magnetic resonance imaging
EMB	Endomyocardial biopsy
PCR	Polymerase chain reaction
